# Vertebral involvement in Erdheim-Chester disease: a case report of non-BRAF-driven diagnosis and treatment challenges

**DOI:** 10.3389/fonc.2026.1785307

**Published:** 2026-04-28

**Authors:** Haocheng Zhao, Weifei Xie, Shanshan Lin, Lingzhi Wu, Wenqiu Wu

**Affiliations:** 1 Department of Hematology, Yueqing People’s Hospital, Yueqing, China; 2 Department of Internal Medicine, Yueqing People’s Hospital, Yueqing, China; 3 Department of Radiology, Yueqing People’s Hospital, Yueqing, China; 4 Department of Pathology, Yueqing People’s Hospital, Yueqing, China

**Keywords:** Erdheim-Chester disease, misdiagnosis, multifocal sclerosis, non-BRAF mutation, vertebral involvement

## Abstract

Erdheim–Chester disease (ECD) is a rare non-Langerhans cell histiocytosis that is frequently misdiagnosed because of its nonspecific systemic symptoms. We report the case of a 73-year-old woman who presented with persistent back pain, abdominal discomfort, and fever. Imaging revealed predominant thoracolumbar vertebral involvement, characterized by multifocal osteosclerosis on computed tomography and abnormal signal intensity with contrast enhancement on magnetic resonance imaging. This radiologic pattern may closely mimic inflammatory disorders, hematologic malignancies, or metastatic disease. Vertebral biopsy established the diagnosis of ECD. Molecular analysis of peripheral blood did not identify actionable driver mutations in the MAPK/PI3K signaling pathways, including *BRAF* V600E, highlighting the diagnostic and therapeutic challenges associated with non–*BRAF*-driven ECD. Despite corticosteroid therapy and supportive management, the disease followed an aggressive clinical course with a poor outcome. This case underscores vertebral and bone marrow–dominant ECD as an underrecognized presentation and emphasizes the importance of integrating histopathology, imaging findings, and molecular profiling to reduce diagnostic delay and inform management when targetable mutations are absent.

## 1 Introduction

**Figure 1:**
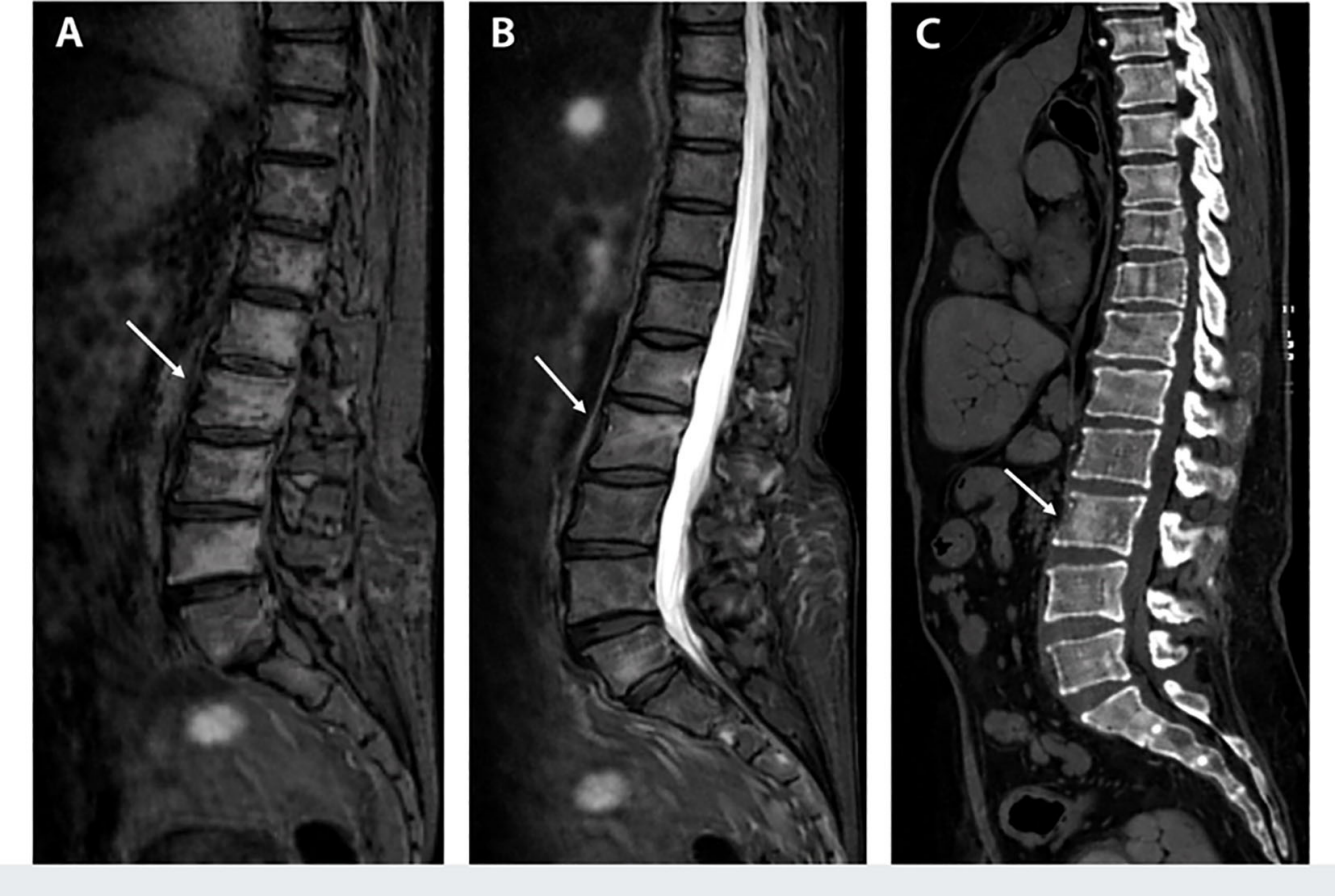
Radiological imaging of the chest and lumbar spine in a patient with Erdheim–Chester disease (ECD). **(A)** Short tau inversion recovery (STIR) magnetic resonance imaging (MRI) showing abnormal signal intensity within the vertebral body and adjacent soft tissues, consistent with infiltrative involvement. **(B)** Contrast-enhanced T1-weighted MRI demonstrating significant enhancement in the affected regions. **(C)** Sagittal computed revealing structural abnormalities of the vertebral column and surrounding tissues, with evident osseous involvement. These imaging findings illustrate the characteristic skeletal and soft-tissue involvement of ECD.

**Figure 2:**
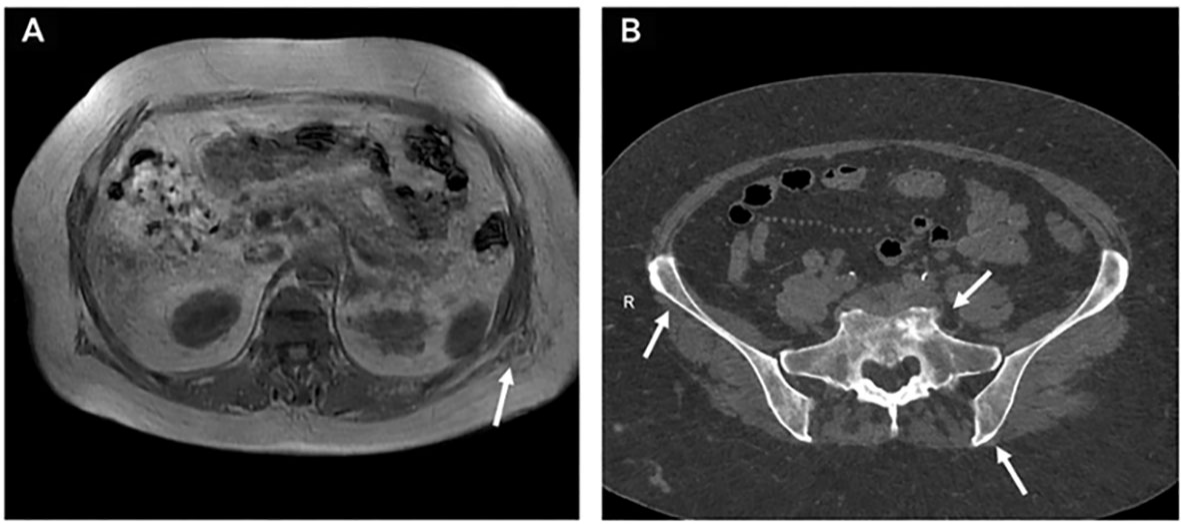
Radiological imaging of skeletal involvement in Erdheim–Chester disease (ECD). **(A)** Sagittal T1-weighted magnetic resonance imaging (MRI) of the lumbar spine showing bilateral cortical sclerosis and abnormal vertebral signal changes characteristic of ECD. **(B)** Contrast-enhanced computed tomography (CT) of the pelvis demonstrating cortical sclerosis, irregular bone remodeling, and cortical thickening, which are typical skeletal features of ECD. This figure highlights the prominent osseous changes observed in ECD, including cortical sclerosis and irregular bone remodeling, which serve as important diagnostic clues.

Erdheim–Chester disease (ECD) is a rare systemic histiocytic disorder characterized by diffuse infiltration of foamy, lipid-laden histiocytes across multiple organs (1, 2). According to the 5th edition of the World Health Organization (WHO) Classification of Hematolymphoid Tumors and the 2022 International Consensus Classification (ICC), Erdheim–Chester disease is categorized among histiocytic and dendritic cell neoplasms, reflecting its clonal myeloid origin and molecular pathogenesis (3, 4). Due to its heterogeneous and nonspecific clinical manifestations, ECD may initially be clinically confused with other hematologic neoplasms, infectious diseases, or metastatic malignancies, often leading to delayed or missed diagnosis (5). At the molecular level, ECD is most commonly driven by activating mutations in the mitogen-activated protein kinase (MAPK) signaling pathway, particularly *BRAF* V600E. However, an increasing number of cases lack *BRAF* mutations and are classified as non–*BRAF*-driven ECD, which further complicates diagnostic evaluation and therapeutic decision-making (1, 6). ECD can involve multiple organ systems, including the skeletal, cardiovascular, retroperitoneal, and central nervous systems (1, 7, 8). Skeletal involvement represents the most prevalent manifestation and classically affects the long bones, presenting as bilateral, symmetric osteosclerosis with the characteristic “hot knees” appearance on radionuclide imaging (9). Cross-sectional imaging modalities, such as magnetic resonance imaging (MRI) and computed tomography (CT), are essential for assessing the extent of organ involvement; however, definitive diagnosis requires histopathological confirmation supported by immunophenotyping and molecular analysis (**Figures 1**, **2**) (5).

**Figure 3:**
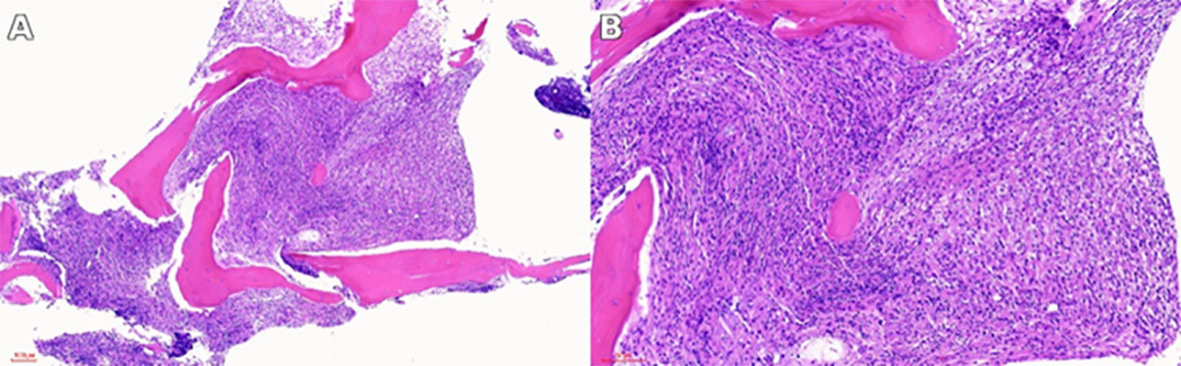
Histopathological examination of vertebral biopsy in Erdheim–Chester disease (ECD). **(A)** Low-power view of hematoxylin and eosin (H&E)–stained vertebral biopsy section showing the overall structure and distribution of the lesion. **(B)** High-power view of H&E staining demonstrating foamy histiocytic cells with abundant pale cytoplasm within a fibrotic background.

**Figure 4:**
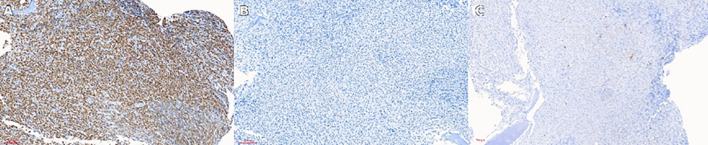
Immunohistochemical staining of vertebral biopsy in Erdheim–Chester disease (ECD). **(A)** Immunohistochemical staining for CD68 showing strong cytoplasmic positivity in histiocytic cells, a hallmark feature of ECD. **(B)** Immunohistochemical staining for CD1a, showing negative expression, aiding in the exclusion of Langerhans cell histiocytosis (LCH). **(C)** Immunohistochemical staining for S-100, showing negative or weak expression, supporting the diagnosis of non-Langerhans cell histiocytic disease. These findings demonstrate the characteristic immunophenotype of ECD, with CD68 positivity confirming histiocytic lineage and CD1a negativity excluding LCH. CD68, cluster of differentiation 68; CD1a, cluster of differentiation 1a; S-100, S100 protein.

In contrast to the typical skeletal pattern, this case describes a 73-year-old woman with ECD predominantly involving the thoracolumbar vertebrae and bone marrow, an uncommon and underrecognized presentation. Imaging revealed multifocal vertebral sclerosis and abnormal signal intensity with contrast enhancement, initially raising suspicion for hematologic malignancy or metastatic disease (**Figures 1**, **2**) (10, 11). Histopathological examination of a vertebral biopsy demonstrated infiltration by foamy histiocytes with characteristic immunohistochemical features, confirming the diagnosis of ECD (**Figures 3**, **4**). Molecular testing failed to identify actionable mutations in the *BRAF* or MAPK/PI3K pathways, underscoring the diagnostic complexity and therapeutic challenges associated with non–*BRAF*-driven ECD. Compared with previously reported cases summarized in the literature (**Table 1**), this case highlights the importance of recognizing vertebral-dominant and bone marrow–predominant ECD, which may be easily overlooked or misclassified as other systemic diseases. Increased awareness of this atypical presentation is essential to facilitate timely diagnosis and appropriate management of this rare condition.

**Table 1:** Clinical profiles and outcomes of representative Erdheim-Chester Disease (ECD) cases.

Author & year	Age/Sex	Organ involvement	Genetics	Treatment	Outcomes
Poellinger (2019) (14)	58/M	Heart, Adrenal, Perir, Bones (Scl)	BRAF V600E (+)	IFN	Prog
Scolaro (2018) (16)	63/M	Kidney, Adrenal, Aorta, RP, Bones	Inconclusive	None	Stab
Benson (2023) (10)	24/M	CNS, Pit (DI), Bones	NR	Ster	Partial Imp
Wilson (2021) (13)	67/M	Bones (Scl), BM (Foamy hist.)	BRAF V600E (+)	NR	NR
Naruse (2010) (17)	31/M	Pit (DI), Joints, Bones (Scl)	LCH overlap	NR	NR
Dai (2022) (15)	29/F	Pancreas, CNS, Bones (Scl)	BRAF V600E (+)	IFN	Imp
Diamond (2016) (8)	68/M	CNS, Pit (DI), Perir, Aorta, Bones	MAP2K1 C121S	Anakinra	Imp
Diamond (2016) (8)	7/M	CNS, Bones (Scl), Facial palsy	BRAF V600E (+)	Anakinra	Imp
Villatoro (2018) (18)	Cohort	Aorta (56%), Heart (61%), Arteries	BRAF V600E (55%)	Clad, IFN, TT	Variable

Data adapted from references (8, 10, 13–18).

BM, bone marrow; Clad, cladribine; CNS, central nervous system; DI, diabetes insipidus; IFN, interferon-alpha; Imp, improved; NR, not reported; Perir, perirenal; Pit, pituitary; Prog, progressed; RP, retroperitoneum; Scl, sclerosis; Stab, stable; Ster, corticosteroids; TT, targeted therapy.

## 2 Case report

A 73-year-old woman was admitted with persistent right upper abdominal pain and progressive lower back pain that had first developed in December 2024 and progressively worsened over the subsequent six months. Over the preceding two weeks, her symptoms had worsened and were accompanied by fatigue, intermittent fever, and night sweats. The lower back pain had gradually worsened over approximately six months and was refractory to conservative management. She also reported vague abdominal discomfort radiating posteriorly. Given the progressive nature of her symptoms, further diagnostic evaluation was initiated. The chronological sequence of clinical events is summarized in **Table 2**.

**Table 2:** Timeline of clinical course.

Date	Clinical event
December 2024	Onset of progressive lower back pain
Dec 2024 – Jun 2025	Evaluated in orthopedic clinic; treated as lumbar strain with NSAIDs; no significant improvement
June 11, 2025	Contrast-enhanced CT performed
June 13, 2025	Hospital admission
June 14, 2025	MRI of thoracolumbar spine
June 19, 2025	Vertebral biopsy and bone marrow evaluation
Late June 2025	Diagnosis of Erdheim–Chester disease established
June–September 2025	Supportive management; patient declined systemic therapy
September 2025	Death due to disease progression

Contrast-enhanced CT of the chest and abdomen revealed multiple cystic lesions in the kidneys and liver, along with diffuse osteosclerotic changes involving the ribs and spine (**Figure 1**). Subsequent MRI of the thoracolumbar spine demonstrated multifocal vertebral sclerosis with abnormal signal intensity and contrast enhancement, findings indicative of a systemic infiltrative or hematologic disorder rather than degenerative disease (**Figure 2**). The imaging findings suggested a diffuse skeletal disorder with vertebral involvement, prompting consideration of ECD in the differential diagnosis. Comprehensive staging evaluation was performed using contrast-enhanced CT of the chest and abdomen and MRI of the thoracolumbar spine. No radiologic evidence of perinephric soft-tissue infiltration (“hairy kidney”) or perivascular sheathing was observed. No cutaneous lesions were identified on physical examination, and no overt cardiac manifestations were clinically apparent during hospitalization. Whole-body PET-CT was recommended for further assessment of systemic disease burden; however, it was not performed because the patient declined referral for additional imaging studies in the context of rapidly progressive clinical deterioration.

Physical examination revealed mild tenderness over the lower lumbar spine without focal neurological deficits or palpable lymphadenopathy. Laboratory testing demonstrated normocytic anemia, with a hemoglobin level of 88 g/L, and elevated inflammatory markers, including C-reactive protein. A vertebral biopsy and bone marrow evaluation were then performed. Histopathological examination showed diffuse infiltration of foamy histiocytes within a fibrotic background (**Figure 3**). Immunohistochemical analysis revealed strong positivity for CD68, with negative staining for CD1a and S-100, consistent with a diagnosis of non-Langerhans cell histiocytosis and ECD (**Figure 4**).

Molecular testing was initially attempted on the formalin-fixed paraffin-embedded vertebral biopsy specimen; however, the tumor cell content did not meet the required threshold for reliable molecular analysis. Due to limited residual tissue following routine histopathological and immunophenotypic evaluation, additional ancillary testing, including BRAF V600E immunohistochemistry, could not be performed. Repeat biopsy was discussed but declined by the patient. Peripheral blood was subsequently analyzed using a targeted next-generation sequencing panel covering recurrent mutations associated with histiocytic neoplasms (12). MTOR, NRAS, and CCND3 mutations were detected in peripheral blood samples. No actionable BRAF V600E mutation or other canonical MAPK pathway driver mutations were identified. These findings indicated a non–*BRAF*-driven molecular profile, limiting the applicability of currently available targeted therapies. Given the absence of actionable mutations, corticosteroid therapy was initiated for symptomatic control. After a detailed discussion with her family, the patient declined further active systemic treatment, including cladribine-based chemotherapy.

Despite supportive management, including analgesic therapy and red blood cell transfusions, the patient’s clinical condition progressively deteriorated, with persistent fever, worsening spinal pain, and progressive anemia. She declined further increase in care and died approximately two months after diagnosis due to disease progression. The clinical presentation, imaging characteristics, molecular findings, treatment strategies, and outcomes of this case are summarized and compared with representative cases of ECD reported in the literature in **Table 1**.

## 3 Discussion

This case highlights an uncommon and diagnostically challenging manifestation of ECD, characterized by predominant vertebral and bone marrow involvement. ECD is a rare systemic non–Langerhans cell histiocytosis with heterogeneous and often nonspecific clinical symptoms, which frequently results in delayed diagnosis or misclassification (1). Skeletal involvement represents a hallmark of ECD and most commonly affects the long bones, where it typically presents as bilateral symmetric osteosclerosis. Axial skeletal involvement, particularly of the bone marrow and vertebrae, is uncommon and remains underrecognized in routine clinical practice (7, 13). In this patient, thoracolumbar vertebral sclerosis with abnormal signal intensity and contrast enhancement on MRI (**Figures 1**, **2**) closely resembled hematologic malignancy or a metastatic disease, highlighting a key diagnostic pitfall in atypical skeletal presentations. Definitive diagnosis, therefore, relied on histopathological confirmation through vertebral biopsy (**Figure 3**), supported by characteristic immunophenotypic findings (**Figure 4**) (5, 14). This case underscores the importance of maintaining a high index of suspicion for ECD in patients presenting with unexplained vertebral sclerosis accompanied by systemic inflammatory features.

The diagnostic complexity observed here is consistent with previous reports describing bone marrow or axial skeletal involvement in ECD, which is frequently confused with plasma cell dyscrasias, myeloproliferative neoplasms, or metastatic malignancies (13). Although the classic “hot knees” sign and long-bone sclerosis are well-established imaging features of ECD, vertebral involvement remains underreported in the literature (8, 10). Previous studies have shown that bone marrow infiltration by foamy histiocytes may present with anemia, systemic inflammation, and nonspecific imaging findings, further complicating timely diagnosis (13). In this case, multifocal vertebral sclerosis with contrast enhancement (**Figure 2**) reinforced the need for tissue biopsy when imaging findings are inconclusive. As summarized in **Table 1**, vertebral or bone marrow involvement is associated with heterogeneous clinical courses and variable outcomes (8, 10, 13–18). Molecular profiling adds another layer of complexity. While *BRAF* V600E mutations are most frequently identified in ECD, an increasing number of patients harbor non–*BRAF* mutations or lack identifiable actionable mutations altogether (6). This molecular heterogeneity contributes to diagnostic uncertainty and significantly limits therapeutic options. In this case, no actionable MAPK pathway driver mutations were identified in peripheral blood samples, and tissue-based molecular profiling could not be completed due to insufficient tumor cell content.

This case expands the recognized clinical spectrum of ECD by illustrating a vertebral-dominant, non–*BRAF*-driven presentation with an aggressive clinical course. Current international consensus recommendations for the diagnosis and management of Erdheim–Chester disease emphasize comprehensive molecular testing, organ-based staging, and targeted therapy guided by MAPK pathway alterations (1). Although current therapeutic paradigms emphasize the central role of *BRAF* mutations in guiding treatment, emerging evidence suggests that non–*BRAF*-driven ECD represents a biologically distinct subgroup associated with fewer treatment options and less favorable outcomes (6). Compared with previously reported cases (**Table 1**), the rapid progression observed in this patient highlights the potential prognostic implications of axial skeletal and bone marrow involvement (13, 18). These findings support integrating comprehensive imaging, early histopathological confirmation, and broad molecular profiling into the diagnostic workflow for suspected ECD (5). The absence of whole-body PET-CT represents a limitation in fully assessing total disease burden and potential subclinical organ involvement. Another limitation of this study is that BRAF V600E immunohistochemistry was not performed on the vertebral biopsy specimen because of limited residual tissue and the inability to obtain repeat biopsy material (19). As a single-case report, this study is inherently limited in its generalizability, and the clinical course and treatment outcome observed here may not be representative of all patients with Erdheim–Chester disease. In addition, although the patient did not receive immunosuppressive or immunotherapy-based treatment and further active systemic therapy was declined by the patient and her family, potential immunomodulatory approaches warrant consideration in the discussion of BRAF-negative ECD. In selected cases, agents such as anakinra have been reported as possible therapeutic options, although evidence remains limited and treatment decisions should be individualized according to molecular findings, organ involvement, disease severity, and overall clinical condition. Improved recognition of atypical skeletal patterns, including vertebral-dominant disease, may facilitate earlier diagnosis and timely referral to specialized centers, which remains critical for optimizing management and outcomes in this rare and heterogeneous disorder.

## 4 Conclusion

This case highlights the importance of including ECD in the differential diagnosis of patients presenting with multisystem involvement and nonspecific systemic symptoms, particularly when atypical skeletal features such as vertebral sclerosis and bone marrow involvement are identified. These uncommon manifestations may closely mimic metastatic disease, hematologic malignancies, or chronic inflammatory conditions, contributing to diagnostic delay. Furthermore, the absence of actionable *BRAF* or MAPK/PI3K pathway mutations in this patient underscores the diagnostic and therapeutic challenges associated with non–*BRAF*-driven ECD and limits the use of currently available targeted therapies (6). This case expands the recognized clinical spectrum of ECD and underscores the need for an integrated diagnostic approach combining imaging findings, histopathological evaluation, and molecular profiling. Increased awareness of atypical presentations and refinement of diagnostic strategies are essential for achieving earlier diagnosis and improving clinical management in this rare and heterogeneous disease.

## Data Availability

The original contributions presented in the study are included in the article/supplementary material. Further inquiries can be directed to the corresponding author.
